# Biological interaction levels of zinc oxide nanoparticles; lettuce seeds as case study

**DOI:** 10.1016/j.heliyon.2020.e03983

**Published:** 2020-05-29

**Authors:** Rabeah Yousef Rawashdeh, Amal Mohammad Harb, Asma Mahmoud AlHasan

**Affiliations:** Department of Biological Sciences, Yarmouk University, Irbid, Jordan

**Keywords:** Nanomaterials, Biological sciences, Materials application, Plant biology, Proteins, Biochemistry, Nanoparticles, Seedling length, Biochemical analysis, Antioxidants, Size

## Abstract

**Background:**

Seed germination is a critical stage in plant life, and recent practices use nanomaterials for the improvement of plant seed germination indices. This study was conducted to assess the effect of laboratory prepared zinc oxide nanoparticles on the physiological and biochemical changes of lettuce seeds.

**Methods:**

Lettuce seeds were soaked in a suspension of moderately polydisperse zinc oxide nanoparticles at two different concentrations (25 ppm or 50 ppm) and shaken for 3 h at 25 °C. Seeds treatment was followed subsequently by two to three days drying at ambient conditions. Treated seeds were stored for 3–4 weeks, at ambient conditions and then tested for germination in petri dishes. Germination was observed on daily basis and seedling length was measured. After imbibition and before the start of the visible germination, seeds were examined for topography and surface analysis using the scanning electron microscope and zinc uptake was measured by using the atomic absorption spectrometry and the energy dispersive X-ray. The pattern of mobilization of biomolecules was analyzed to detect any differences among different seed groups.

**Results:**

There was no loss of viability for the nanoparticles treated seeds. Indeed their germination was enhanced and their biomass increased. The activated performance of the nanoparticles imbibed seeds has been found to be correlated with an increased level of Zn inside lettuce seeds. The recorded measurements show a significant enhancement of seedling length. Interaction of zinc oxide nanoparticles with lettuce seeds mediates a variation in the biochemical processes. Changes detected in treated seeds were as following: reduced levels of the total carbohydrates (including simple saccharides and polysaccharides), higher capacity of protein synthesis, an elevated level of starch as well as an increased activity of antioxidant enzymes.

**Discussion and conclusion:**

Lettuce seeds primed with ZnO nanoparticles were found not only to maintain seed viability but even to exhibit a detectable level of germination enhancement compared to the control seeds. Overall, the promoted response of lettuce seeds during early stages of seed growth is encouraging for the application of ZnO NPs for seed priming for better germination indices.

## 1 Introduction

Nanoparticles have been vastly explored for biomedical applications such as; biocides agents (Hemeg, 2017), gene therapy (Liu and Zhang, 2011), cellular imaging (Hemmerich and von Mikecz, 2013) and molecular recognition and sensing (Yan and Chen, 2019). Useful agricultural applications have been reported in the literature as well, such as; the use of nanoparticles for enhancing plant growth and yield (Karny et al., 2018; Rastogi et al., 2019; Sanzari et al., 2019).

Despite the concerns about their toxicity and side effects, the negative impacts of many nanoparticles can be limited by a careful control of their application conditions and parameters (Zalk et al., 2009; Beck-Broichsitter et al., 2012; Brohi et al., 2017; Hemeg, 2017; Gupta and Xie, 2018). The step wise assessment starts by observing the exerted effects of nanoparticles on living subjects such as plants (Kumari et al., 2012). The desirable and undesirable biological effects of NPs are excreted as a result of the uptake, penetration, absorption, adsorption, oxidation and accumulation in the targeted biological subject (Pérez-de-Luque, 2017; Lv et al., 2019).

There is a critical need for a comprehensive examination of the inducible metabolic activities of the efficient nanomaterials (Sharpe et al., 2011; Velusamy et al., 2016; Rawat et al., 2018; Sanzari et al., 2019). A variety of factors impact the mode of action of nanoparticles (NPs), and one fit hypothesis is not applicable for the mechanisms of NPs. Parameters such as: the concentration, the solvent, the stability of NPs, as well as the physiochemical characteristics of nanomaterials impact their behavior in biological systems (Wang et al., 2017; Rastogi et al., 2019). and hence, their mechanism of action is mainly based on the experimental conditions (Rawashdeh and Haik, 2009).

Beside the fact that several studies observed no toxic effect of nanoparticles, their beneficial impact on plant growth and development was demonstrated by many previous studies (Nair et al., 2011; Pereira et al., 2019; Rastogi et al., 2019). Metal and metal oxide nanoparticles of titanium dioxide, silicon dioxide, silver, sulfur, zinc oxide were tested for enhancing seed germination and plant growth of Canola (Mahmoodzadeh et al., 2013), tomato (Siddiqui and Al-Whaibi. 2014), zucchini (Almutairi and Alharbi, 2015), cucumber (Al-Banna et al., 2016), and chili pepper (García-López et al., 2018) in the respective order. However the cellular and molecular mechanisms of NPs were not specifically illustrated or described by many of the scientific articles (Sanzari et al., 2019).

Conventional seed priming is a common practice that involves seed soaking in water or solution for a few hours to break seed dormancy and to improve germination and seedling capacity (Chen and Arora, 2013). Nano priming is advantageous over the conventional hydropriming, and it results in significant enhancement of seed germination and plant growth in different plant species (Rosa et al., 2013; Abou-Zeid and Moustafa, 2014; Acharya et al., 2019).

There are different routes of nano metals internalization inside plant cells and the main ones are by endocytosis, pore formation and or by a controlled release of ions (Mahakham et al., 2017; Pérez-de-Luque, 2017; Juárez-Maldonado et al., 2019; Sanzari et al., 2019). Metallic NPs have cumulative ion release compared to a constant supply of ions from their bulky counterparts. The former pattern of ion release is more efficient because it provides a continuous leaking of ions from particles (Xu et al., 2018).

Pretreatment of plant seeds with nano sized metals provides an alternative to the utilization of metals containing fertilizer that are necessary for plant growth (Siddiqui and Al-Whaibi. 2014). Some reports demonstrated that the use of NPs for treating seeds is more efficient and advantageous over traditional treatment utilizing hormones and fertilizers in terms of plant yield and quality (Pereira et al., 2017). Metals at nano scale have better kinetics and biological interaction. Zinc is an essential metal that is critical for plant growth and its role in early stage via seed germination is not less important.

Lettuce seeds were used in the present study as a model to investigate the effect of NPs on the biochemical activities of the germinating seeds. The main objectives of this study are to test the effect of zinc oxide nanoparticles on seed germination of lettuce and to evaluate the morphological and biochemical changes in the nano-treated seeds during their germination.

## 2 Materials & methods

### 2.1 Dispersion of nanoparticle colloidal suspension and nanoparticles characterization

ZnO NP powder was obtained from the Nano-center at Jordan university of Science and Technology (JUST). ZnO NPs stock colloidal suspension was prepared by dispersing 0.01g of ZnO powder in 10 ml sterile distilled water and the suspension was sonicated for 20 min (by Ultrasonic cleaner WUC-D06H Daihan Scientific Co Ltd - S.Korea). Sterile distilled water was used to dilute the stock suspension of ZnO NPs to concentrations of: 25 ppm and 50 ppm. NPs stock and diluted suspension were freshly prepared.

Nanoparticles suspensions were characterized using: the Scanning Electron Microscope with Energy Dispersive X rays (EDX), which was carried out using SEM (SEM, Quanta 450 FEG, FEI, Hillsboro, OR, USA). The SEM was done at a 10- to 30-kV acceleration voltage. Imaging was detected at 8 mm working distance. EDX technique was employed to determine the composition of chemical elements in ZnO nanoparticles. Further routine characterization was done using; UV-VIS spectrophotometer and Zeta potential meter (Malvern Instruments, Zeta sizer Nano ZS 90).

### 2.2 Seed treatment

Lettuce seeds used in this experiment were bought from a local agricultural store (Irbid, Jordan). These seeds were produced by Larosa Emanuele Company (Larosa Emanuele SEEDS Company, Andria, Italy). Seeds were used in nanopriming without prior sterilization.

Two concentrations of ZnO NPs (25 and 50 ppm) were tested for lettuce seed germination. Healthy lettuce seeds were chosen for each of the following treatments: 0 μg/ml ZnO NPs (control), 25 μg/ml ZnO NPs, 50 μg/ml ZNO NPs. The selected seeds were mixed with 50 ml of each of the prepared concentration of NPs suspension and incubated with shaking for 3 h at 25 °C in dark. After NPs treatment, seeds were divided into two groups: the first group had 50 seeds that were transferred to a 15 mm petri dish and then dried for 2–3 days at ambient conditions. The second group had the remaining 20 seeds that were placed on aluminum foil for oven drying at 80 °C for 3 days. The weight was taken before and after oven drying to estimate the water content according to the following equation:

Water content % = (Wi – Wf)/Wi∗100%

Wi: initial weight, Wf: dried weight

### 2.3 SEM imaging of seeds surfaces and quantitative analysis of Zn uptake by seeds

The dried seeds were mounted on stubs using conductive double-sided carbon tape and sputter coated with a thin layer of gold before SEM imaging. The energy dispersive x rays (EDX) technique was used to validate the elemental weight percentage in lettuce seeds and to verify the relative proportions of elements in different seed samples. Zinc concentration in NPs suspensions was detected using flame Atomic absorption spectrometry (AAS) by Perkin Elmer AAnalyst 200 model (Mundelein, Illinois USA). Two ZnO NPs suspensions at different concentrations: 25 ppm and 50 ppm were tested for zinc ions release. ZnO NPs suspension used for seed treatment was prepared for analysis as following; after 3 h incubation, centrifugation was done at 2000r/m for 4 min to precipitate seeds and the supernatant was transferred to a new tube for AAS measurement. Percentage change in Zn concentration was calculated according to the following equation:

Change in [Zn] = ([Zn]f – [Zn]i)/[Zn]i ∗ 100

[Zn]i: initial concentration; [Zn]f: final concentration

### 2.4 Seed germination

Primed seeds were stored for a period of 3–4 weeks at ambient conditions before they were set to germinate in petri dishes. Germination was done according to Siddiqui and Al-Whaibi (2014) with some modifications. The first group of 50 seeds from each concentration treatment was placed onto a filter paper in a 15 cm Petri dish. The filter paper was moistened with 6 ml of sterile distilled water. The plates were sealed with parafilm and placed inside the incubator at 25 °C. After 2 days of incubation, seeds started to germinate and germination was monitored over 5 days, until day 7 of incubation, by daily recording the number of the germinated seeds in each group. Additionally, seedling length of the grown seeds was measured after 5 days.

The calculation of the following; germination percentage, mean germination time, and mean germination rate was done using the following equations shown in the following table:

**Table:** 

G % Germination percentage	$G\%=\frac{\sum_{i=1}^{k}\text{ni}}{\text{N}}x100$
MGT (day) Mean germination time	$t=\frac{\sum_{i=1}^{k}\text{ni}\;\text{ti}}{\sum_{i=1}^{k}\text{nt}}$
MGR Mean germination rate	$v=\frac{\text{i}}{\text{t}}$

• ni = germinated seed per day i, ti = day of germination, nt = total germinated seeds,
• Seedlings length and fresh weight were taken at the end of the experiment

### 2.5 Biochemical analysis of seeds storage during imbibition

Seed extract was prepared according to a modified method of Samad et al. (2013) as following; 1 g of seeds from each concentration was placed in a 15 ml tube and 2.3 ml of distilled water was added in each tube. Tubes were placed in water bath for 3 h at 90 °C. The last step was repeated twice and the extract was stored at -20 °C. Seed extracts were subjected to biochemical analysis for the quantitation of sugar and the total protein and for the detection of the activities of the amylases and the catalases. The Thermo Scientific Multiskan GO UV/V microplate spectrophotometer (Thermo Fisher Scientific Oy. Ratastie 2, FINLAND) was used for all spectrophotometric assays of carbohydrates, proteins and enzymes.

### 2.6 Quantification of total carbohydrates, proteins and starch

Carbohydrates were quantified by using total carbohydrate assay kit following the manufacturer's instructions (ab 155891. Abcam. UK). Total protein content (mg/ml) was quantified according to Bradford's method (1976). Briefly; 1mg of Coomassie Blue G250 was dissolved in 50 ml 95% ethanol, and then 100 ml 85% phosphoric acid. Dye was diluted 1:5 and then filtered using A4 Whatman filter paper. A 0.002 g of Bovine serum Albumin protein (BSA) was dissolved in 1 ml of PBS (phosphate buffer saline) to get 2 mg/ml protein concentration. A range of concentrations of BSA (range 0.0–1.6 mg/ml) was added into wells of 96 plate, followed by the addition of 5 μl of unknown sample. A 250 μl of diluted, filtered Bradford reagent was added then the plate was incubated for 15 min and absorbance was read at 595nm.

Measurement of starch levels (mg/ml) was carried out in a microplate according to Xiao et al. (2006). The total reaction volume of the starch iodine test was 200 μl, and it was conducted as following; 40 μl of 0.02 M sodium phosphate buffer (pH 6.9 containing 6 mM sodium chloride) was added to each well, 20 μl of the test samples were added and incubated at 37 °C for 10 min. 20 μl of the soluble starch (1%, w/v) was added to each reaction well and incubated at 37 °C for 15 min. 20 μl of 1 M HCl was added to stop the reaction, followed by the addition of 100 μl of iodine reagent (5 mM I_2_ and 5 mM KI). The development of a dark blue color indicates the presence of starch while a yellow color indicates the absence of starch. The absorbance was measured at 620 nm using the microplate reader.

### 2.7 Determination of the activity of beta-amylases and catalases

The activity of beta-amylases was run according to the manufacturer's instructions (General Beta amylase kit, BMY1 assay kit. MyBioSource, Inc., San Diego, CA. USA). The activity of catalase was determined using the CAT assay kit (catalase kit MBS2548470. MyBioSource, Inc., San Diego, CA. USA). The activity of catalase was detected by measuring the reduction of H_2_O_2_ at 240nm.

### 2.8 Statistical analysis of experimental data

The experimental research was designed to have three nanoparticle treatment groups; First group was with no treatment (zero NPs concentration), second group was treated with 25 ppm and the third group was treated with 50 ppm. SPSS software (SPSS Statistics for Windows, version 22.0 (IBM Corp., Armonk, N.Y., USA) was used in this study, the mathematical functions used are mean *M*, median and mean rank to explain the statistical concepts and for the descriptive analysis. Relative graphical representations were documented as well.

One way ANOVA was used to explain the mean *M* differences among the three groups of the study. The alpha level was set less than 0.05 for all statistical tests (p < 0.05). ANOVA was used because distributions are not heavily skewed. ANOVA analyses with follow-up tests were conducted to evaluate pairwise differences among the means. Tukey's HSD procedure was used for conducting post hoc method comparison.

## 3 Results and discussion

### 3.1 Characterization of ZnO NPs

**Figure 1:**
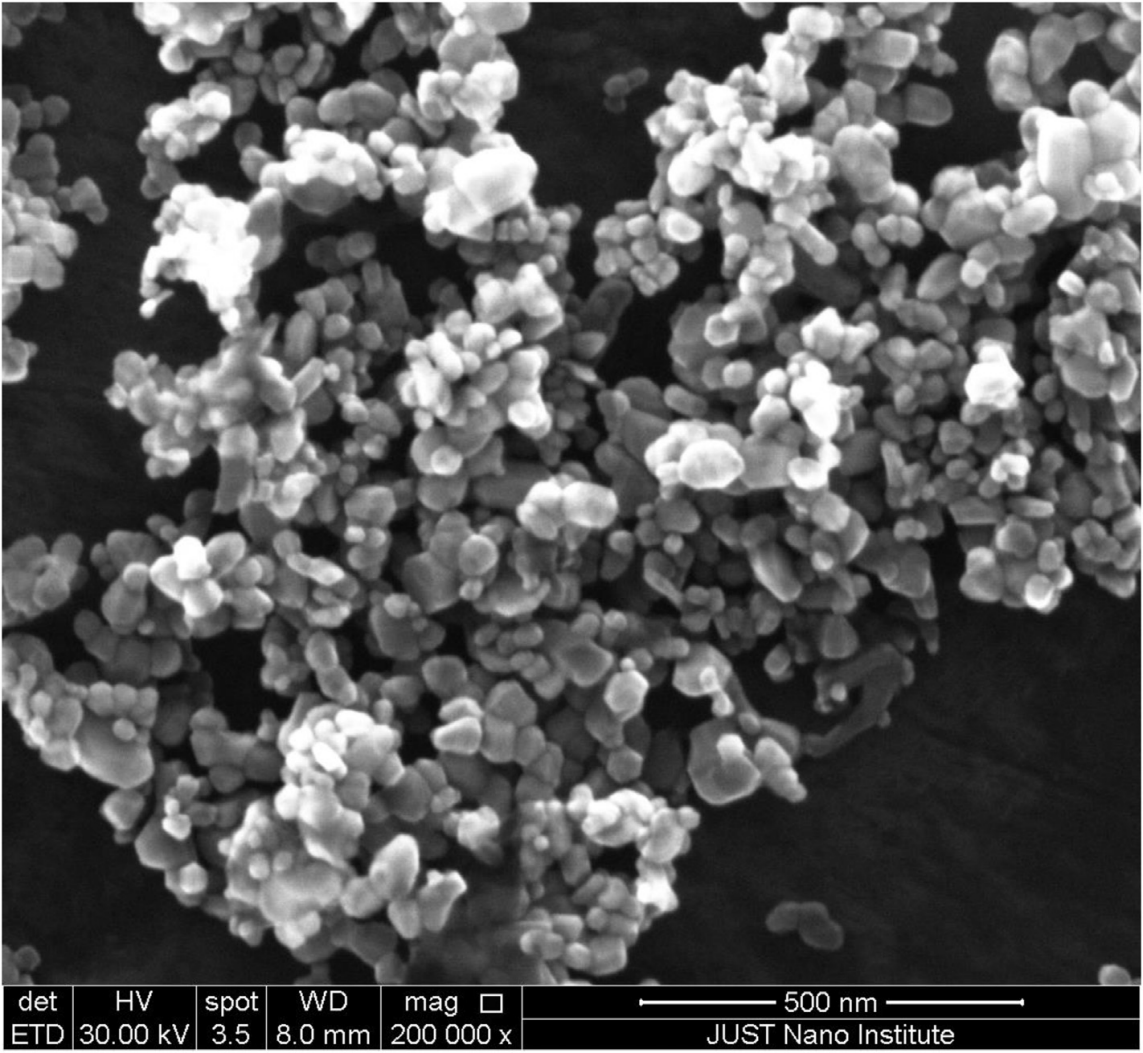
SEM image of Zinc oxide nanoparticles.

The presence of Zinc oxide nanoparticles was confirmed in the suspension by using UV visible absorbance spectroscopy, the detection of an absorbance peak at 380 nm correlates to the presence of ZnO NPs. The spectrum is available as supplementary material (Fig. S1). Nanoparticles size was examined using the scanning electron microscope (SEM) (Figure 1). The average size of individual nanoparticles is approximately 50 nm, however there is a large agglomeration of nanoparticles; size of aggregates around 1000 nm. Energy dispersive x rays (EDX) technique was employed to determine the relative elemental composition in ZnO nanoparticles by measuring the intensity of the characteristic emitted X rays. EDX confirmed the presence of only the two elements; zinc and oxygen (80% of Zinc and 19% of oxygen) in zinc oxide nanoparticle. The analytical data are available as supplementary material (Fig. S2). EDX analysis proved the purity of the obtained samples of ZnO NPs and our data are in accordance with the results of similar research (Raj and AJayalakshmy, 2015).

Zeta potential measurement was recorded in the negative range from -20 to -23 mV, the polydispersity index (PDI) is commonly measured by using dynamic light scattering (DLS) and in this study a low PDI index was recorded (PdI <0.2) indicating a moderate particle size distribution (Hughes et al., 2015). The hydrodynamic radius of a given particle in solution was recorded in the range 700–1200 nm indicating the agglomeration of the particles as a result of the Brownian motion.

Zeta potential is a property of interfacial layer and it is usually measured to indicate the stability of NPs in medium (Shao et al., 2015). Surface charge is important for assessing the stability of the particles in a medium. The stronger the charge the better is the stability of the particles and the longer the shelf life is. Zeta potential is affected not only by the properties of nanoparticles, but also the nature of the solution, such as pH and ionic strength. The nanoparticle can attain different overall charge based on interactions with the surrounding medium. Distilled water used had a slightly increased pH, which most likely increased the ionic stabilization of the NPs suspension.

### 3.2 Seed germination

Lettuce seeds that were soaked in NPs, subsequently were dried for 2–3 days under ambient conditions. The imbibed seeds were germinated, and the visible protrusion of the radicle is the starting sign of the visible germination. Seed populations have different germination percentage and speed. Table 1 shows the important parameters estimated from lettuce seed germination.

**Table 1:** Descriptive analysis of lettuce seed germination parameters. Lettuce seeds were treated with different ZnO NPs concentrations: 0 ppm, 25 ppm and 50 ppm.

Germination Index	Concentration of NPs ppm	*M* + SD∗	Range
Number of Germinated seeds	0.0	40.67 ± 3.076795	37–44
	25	39.167 ± 3.4303	36–44
	50	41.67 ± 4.179	35–47
Germination rate	0.0	0.3133 ± 0.02178	0.27–0.333
	25	0.331 ± 0.00195	0.328–0.333
	50	0.32145 ± 0.0133	0.295–0.331
Germination percentage	0.0	81.33 ± 6.15	74–88
	25	78.33 ± 6.86	72–88
	50	83.33 ± 8.358	70–94
Germination time	0.0	3.205 ± 0.238	3.0–3.19
	25	3.021 ± 0.0178	3.0–3.045
	50	3.116 ± 0.137	3.02–3.386
Water content	0.0	27.75 ± 15.458	15.0–50
	25	18.33 ± 14.5	5.0–37
	50	27.33 ± 23.00	0.0–52

∗ *M* = mean. *SD* = standard Deviation. Mean values were calculated for a sample size of 6 plates for each group of seeds, and in each plate there were 50 seeds.

**Figure 2:**
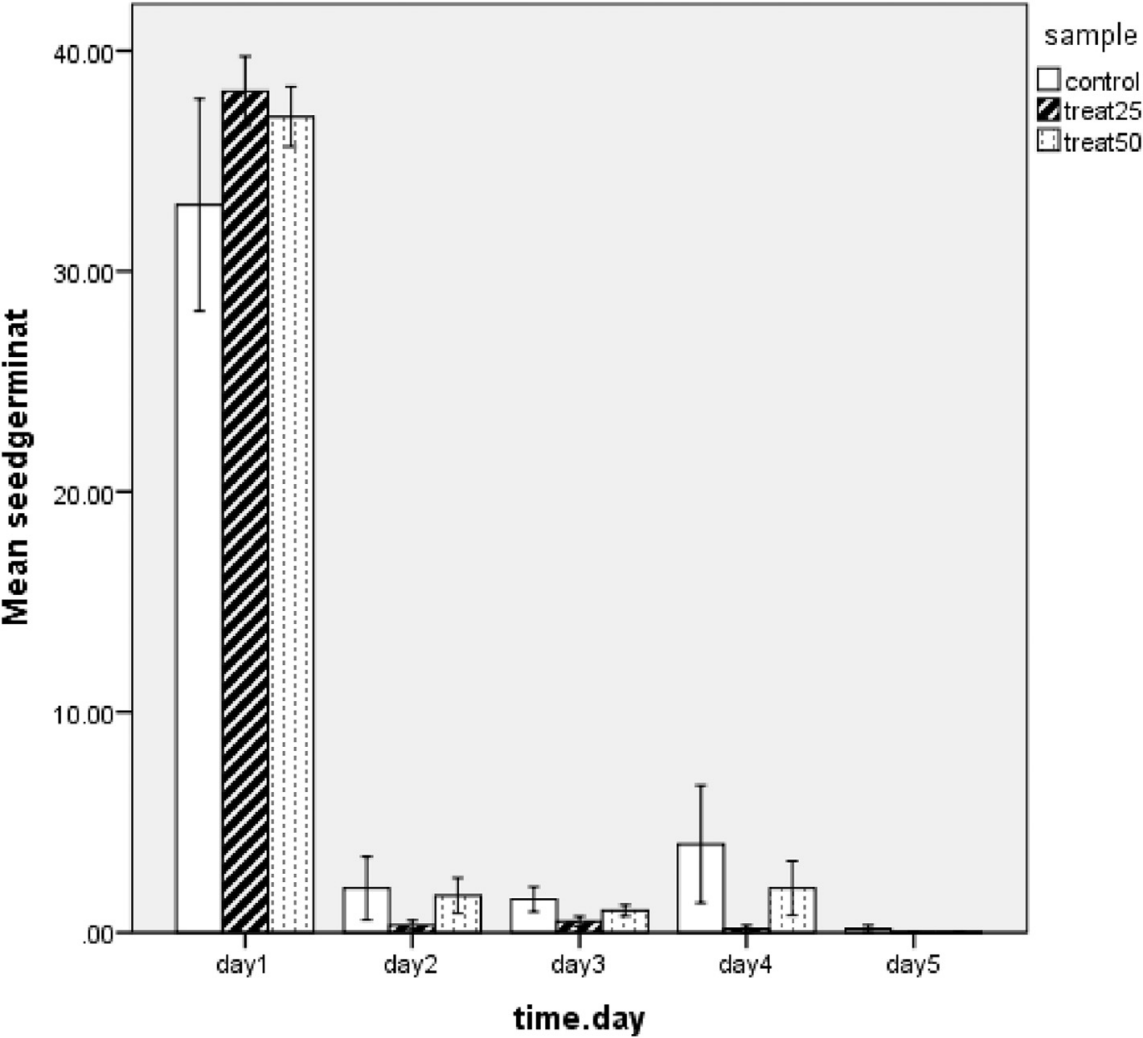
Mean values of the number of germinated seeds over time after lettuce seeds treatment with ZnO NPs using 25 ppm and 50 ppm. The mean values of germinated seeds were calculated for a total of 300 seeds distributed into 6 plates (N = 6 plates) for each group.

For data distribution of seed germination among the three groups, ONE Way ANOVA for the test is not significant F (2, 87) = 0.009, p = 0.991. Measurements of seed germination were taken over time there are 6 plates for each group over five time points, statistical analysis did not detect a significant difference between groups in seed germination among groups over time (Figure 2). Distribution of any time point is the same across the three groups (ANOVA F (14, 75) = 1.34, p = 0.311).

**Figure 3:**
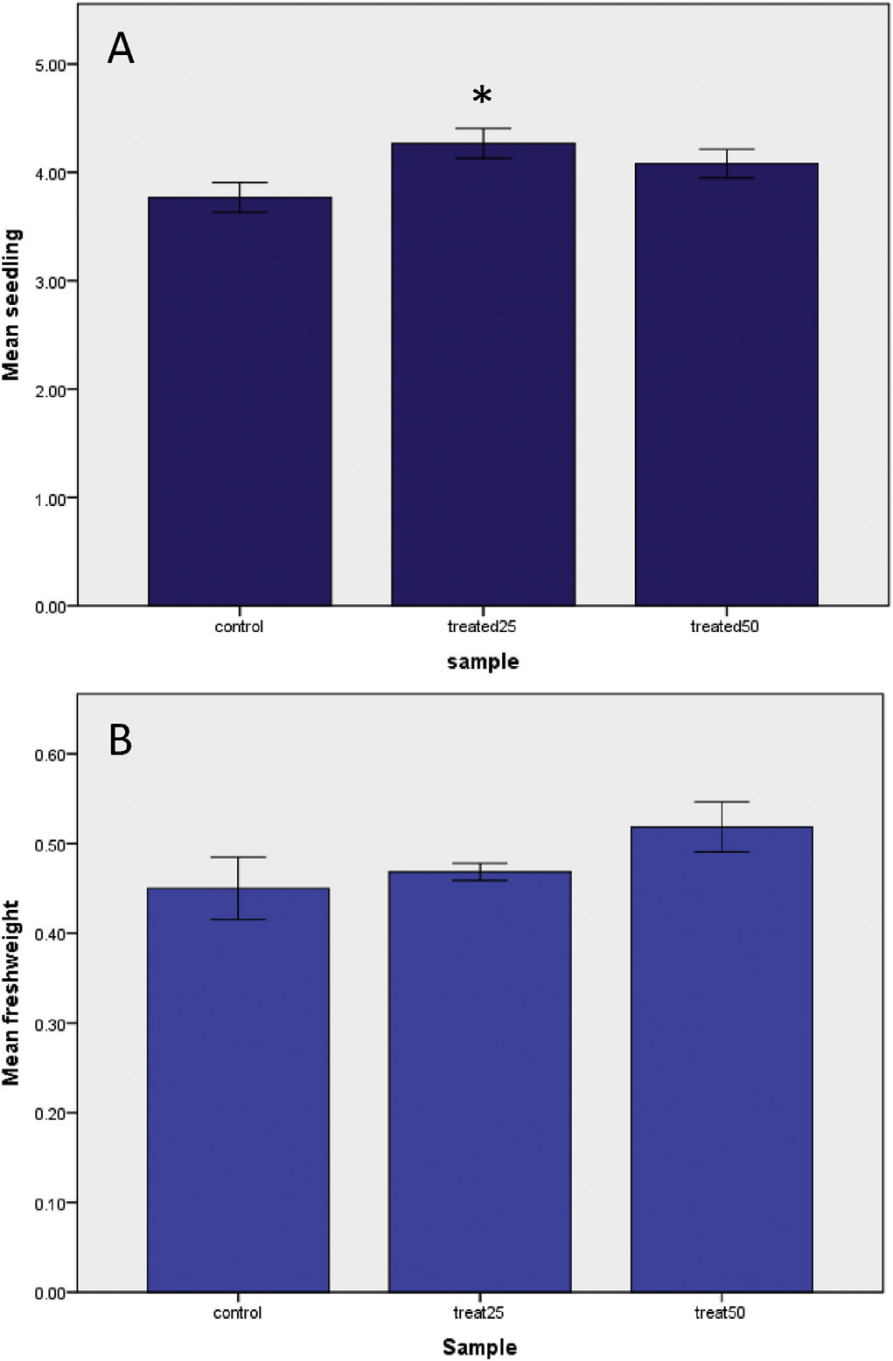
Effect of two different concentrations of ZnO NPs (25 ppm and 50 ppm) on both lettuce seedling length (A) and fresh weight of lettuce seedling (B). Values are the means for each criterion and the vertical bars represent standard errors. Significant difference (p < 0.05) are marked with “∗”.

Mean differences of seedling length is significant across groups (ANOVA for the test is significant F (2, 807) = 3.321, p = 0.037). ANOVA post hoc comparisons (Tukey's HSD test) state that there was a significant difference in the mean of seedling length between seed group treated with 25 ppm NPs and the control group (p = 0.029, LSD; p = 0.011) (Figure 3, A), but the mean differences is not significant between the 50 ppm NPs group and the control group. Using ANOVA for analysis of fresh weight distribution across the groups (Figure 3, B), there was no significant differences (ANOVA, F (2, 15) = 1.805, P = 0.198).

Mean of germination rate is not significantly different across groups (ANOVA, F (2, 15) = 2.139, p = 0.152). No significant difference in germination percentage was detected ANOVA, F (2, 15) = 0.736, P = 0.495). Mean differences in germination time is not significant across the three groups (ANOVA, F (2, 15) = 2.009, P = 0.169. No differences in water content across groups (ANOVA, F (2, 15) = 0.521, p = 0.604).

Over all, the results of germination are in agreement with other studies (Nair et al., 2011; Pereira et al., 2019). Lettuce seeds treated with NPs were obviously healthy and exhibited no observable toxic effect. In addition to that, growth parameters of seed weight and seedling length were efficiently improved.

### 3.3 SEM imaging of seeds surfaces and quantitative analysis of Zn uptake by seeds

**Figure 4:**
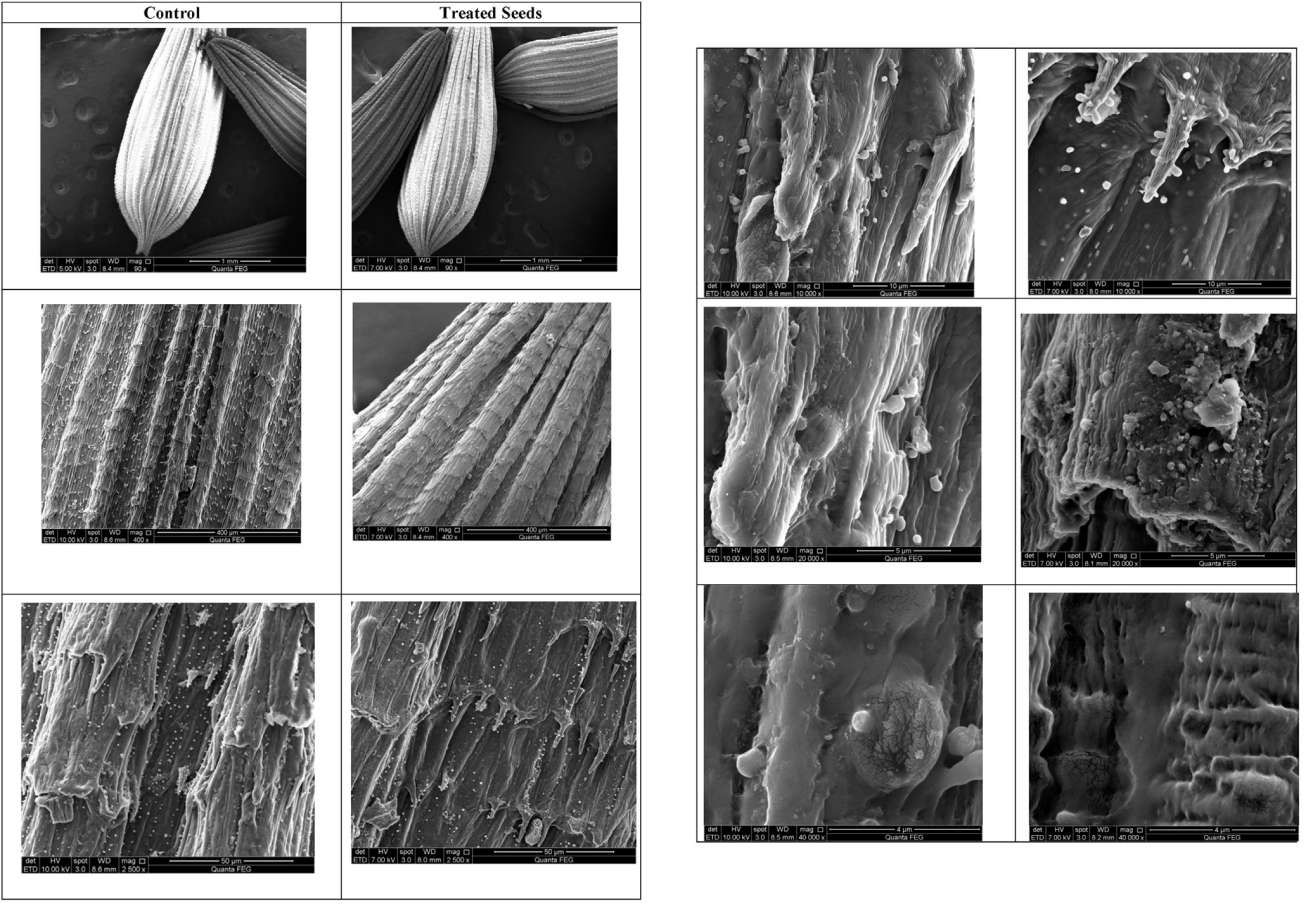
Scanning electron microscope examination of the surface and appearance of lettuce seeds. 1. Low to high magnification of untreated vs. treated. Left Panel: untreated seeds, Right Panel: seed treated with 25 μg/ml ZnO NPs.

The high resolution SEM images displayed no seed surface deformations (Figure 4). There were no collapsed areas or large breaks on the surface of seed coat. Based on the scanning electric microscopic examination, no damage occurred to seed coat as the unbroken surface of treated seeds appeared with no cracks or depressed areas, suggesting biocompatibility and lack of toxicity of NPs. Pits formation increases membrane permeability and allows for metal depletion which eventually causes cell lysis. However surface features of non treated seeds are the same as that of treated seeds, and the surface morphological similarities reflect that there was no excreted physical effect on the surface of seeds induced by nanoparticles. SEM images of this study indicate that the mode of action of NPS was not through pore formation in seed coat.

**Figure 5:**
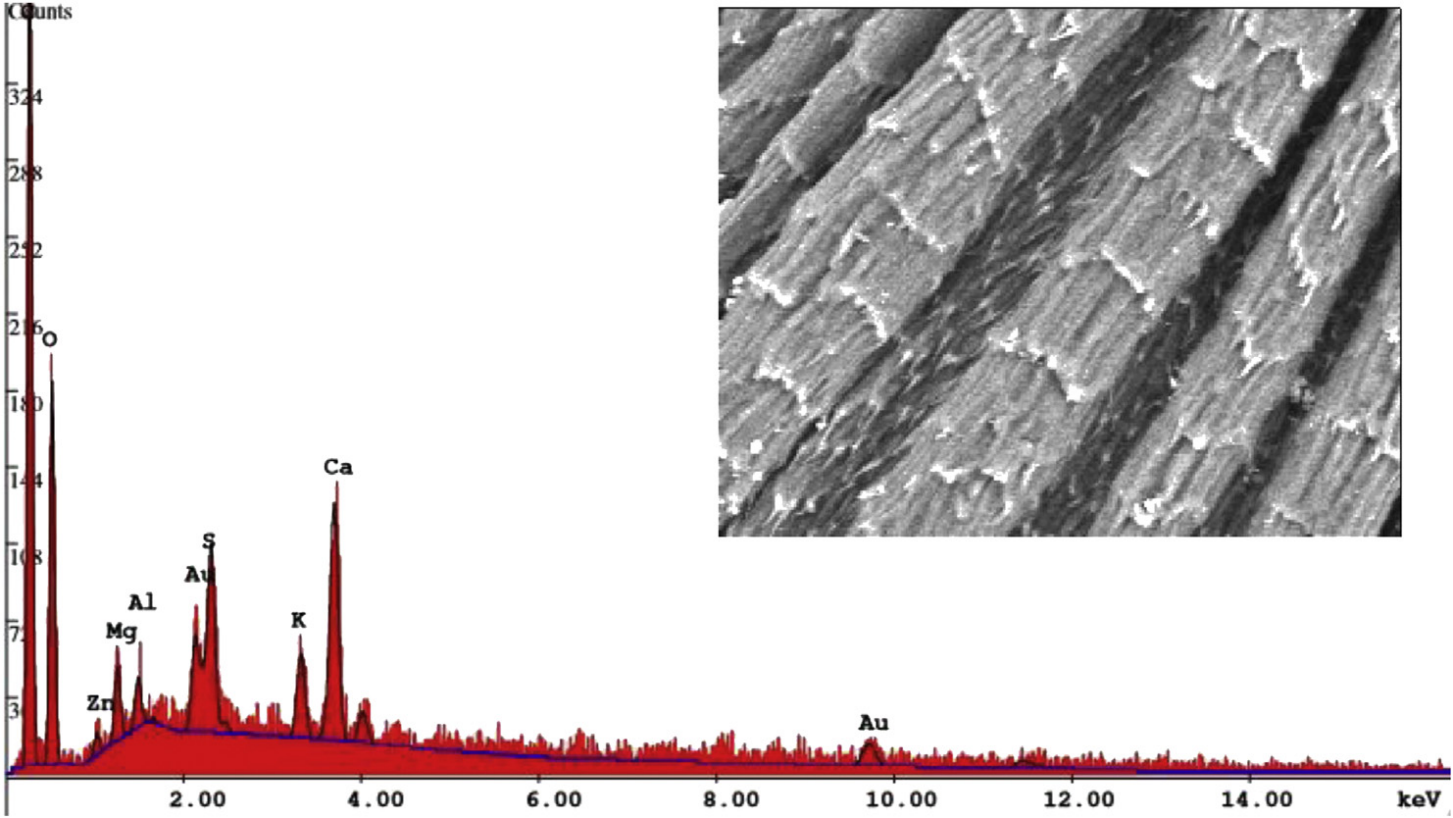
SEM image right-hand side panel and EDX spectra left-hand side panels of lettuce seed treated with 25 ppm ZnO NPs. The EDX spectra sampled from SEM areas. EDX spectrum shows Zn peak of atomic zinc origin ZnL. Prominent peaks were detected;. C, O, Mg, Al, S, K, Ca, denote corresponding chemical elements. The ending letters K and L denote the characteristic binding energies of the electron shells.

A SEM area was scanned by EDX to generate an X-ray spectrum. This quantitative analysis was done to verify the relative proportions of zinc in different seed samples. Figure 5 shows a secondary electron image of a seed specimen and the corresponding X-ray spectra that was generated from the entire scan area. The Y-axis shows the counts (number of X-rays received and processed by the detector) and the X-axis shows the energy level of those counts. Integral x-ray spectra sampled at two sites of interest shows ONE peak of atomic zinc origin ZnL (1.3%). Zn was detected in lower weight percentage (<0.7%) or was not detected in lettuce seeds not treated with NPs (Data not shown for the control seeds). The variation in the relative proportions of zinc in lettuce seeds is attributed to absorption of zinc as a result of NPS treatment (Mahajan et al., 2011).

Atomic Absorption Spectroscopy is a very common technique for the quantitative detection of metal ions and in this study we used AAS for measuring ZnO NPs dissolution after 3 h incubation. Table 2 shows the concentrations of zinc ions released and it is obvious that ZnO NPs suspension released tiny amounts of ions upon dissolving in water.

**Table 2:** The concentration of zinc ions released was measured in ZnO NPs suspension in the absence and presence of lettuce seeds.

ZnO NPs Concentration	No seeds		Seeds	
	Zn^+2^	Zn^+2^ released of the total Zn	Zn^+2^	Zn^+2^ released of the total Zn
25 ppm	0.643	2.572	0.394	1.576
50 ppm	1.08	2.1	0.681	1.362

For elemental analysis, the measured amounts of Zn was reported in ppm (1 ppm equates to ppm).

The release of ions by the dissolved metallic nanoparticles depends on many factors including: pH of the medium, concentrations of the dissolved nanoparticles and the particle size (Liu and Hurt, 2010: Sotiriou et al., 2012).

Zinc ion released from ZnO for the 25 ppm and 50 ppm of ZnO NPs was respectively as following; 2.572% and 2.1% of the total Zn. Thus, the concentration of 25 ppm of NPs was more efficient in releasing ions compared to a concentration of 50 ppm of NPs (2.572% vs. 2.1%). Low concentration of NP suspension demonstrated good dispersion compared than higher NPs suspension. According to other studies, A high concentration of Zn nanoparticles release less zinc ions upon dispersion (Sotiriou and Pratsinis. 2010).

Further AAS analysis was done for the comparison of the detected Zn levels in nanoparticles suspension used for treating seeds. Zinc concentration in NPs suspensions was reduced after seed treatment. Seed absorbed 0.738 of Zn ions released from 50 ppm. In contrast to the released amount of 0.996 zinc ions absorbed by seeds treated with 25 ppm.

On the basis of AAS results along with EDX measurement it can be suggested that the imbibed seeds had higher zinc content. The entry route of Zn ions was cross seed coat and their transport mechanism was clearly not via pore formation but through increasing coat permeability.

### 3.4 Biochemical analysis

**Figure 6:**
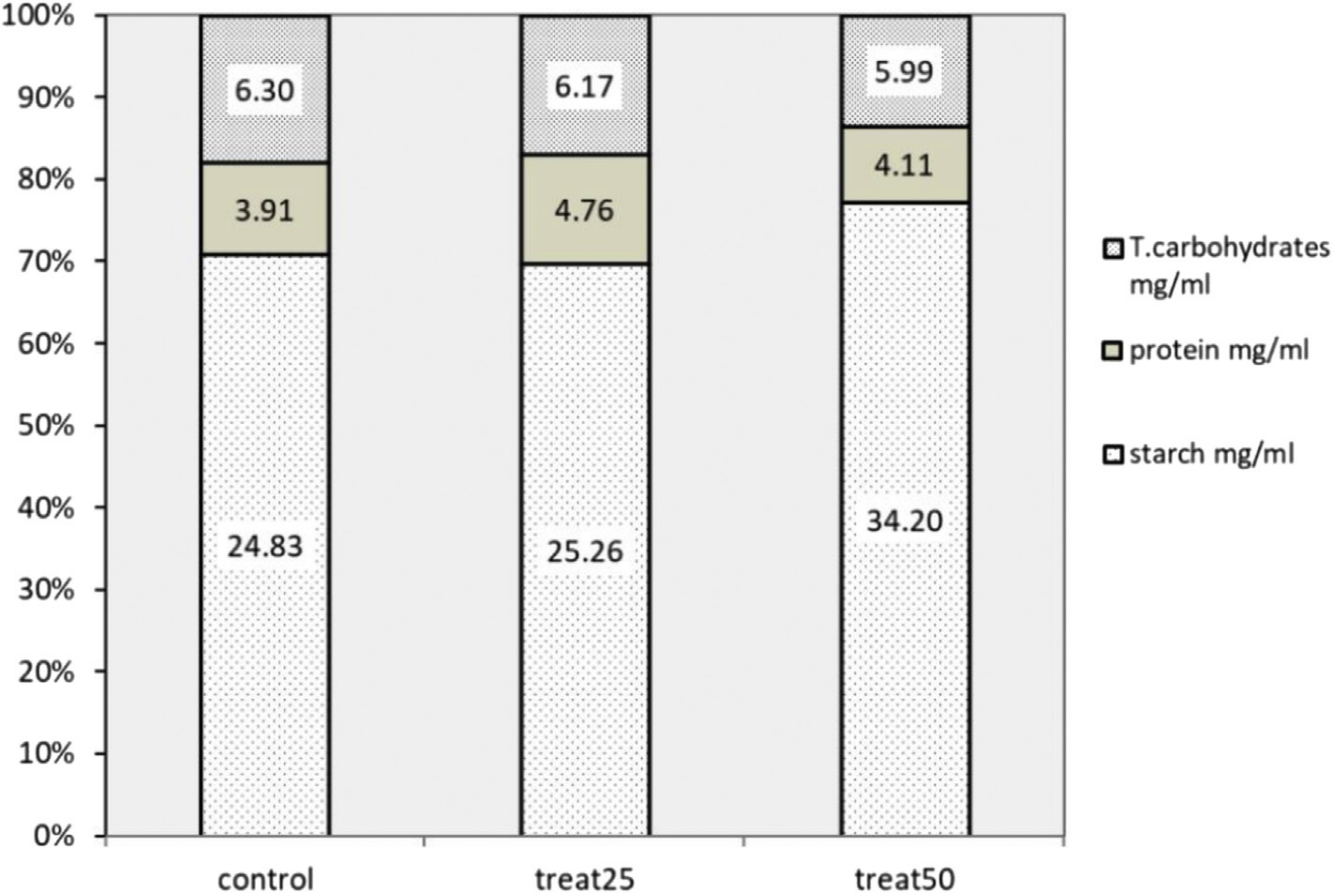
The effect of two concentrations of ZnO NPs; 25 ppm and 50 ppm on the relative proportions of starch, protein and carbohydrates in lettuce seeds. The total of each bar is 100 and the measured concentrations of each of the following components; starch, protein and total carbohydrates are expressed as percentage of the respective totals. Labels inside the rectangle bars represent the measured concentrations (mg/ml) of biomolecules in each seed group.

Certain biochemical changes took place in lettuce seeds during early stages of germination, starting from the onset of 3 h imbibition in ZnO NPs suspension to the subsequent 2 days drying at ambient condition. The dry and the invisibly germinated lettuce seeds were subjected to a heating extraction technique to isolate the bioactive compounds and examined for the total carbohydrate, protein and starch content. The Percentage stacked bar (Figure 6) for the biochemical analysis of seeds extract revealed that the total protein level was higher in treated seeds. The level percentage of protein was >13% in seeds treated with 25 ppm compared to 11% in untreated seeds. The higher protein levels detected in NPs imbibed seeds suggests initiation of *de novo* synthesis of enzymes, during imbibition and early germination, to accommodate metabolic requirements in treated seeds as stated in literature (Wang et al., 2007).

The Percentage stacked bar (Figure 6) for the biochemical analysis of seeds extract revealed a reduction in the total carbohydrate levels in NPs treated seeds (17% in seeds treated with 25 ppm and 13.5% in seeds treated with 50 ppm compared to the untreated seeds (18%). If the interpretation is to be restricted solely on the outcome of the total carbohydrate, it suggests that NPs treatment induced early carbohydrate mobilization that resulted in reduction of the carbohydrate content.

Conversely to the results of the total carbohydrate test, starch iodine test shows higher starch content for NPs treated seeds (Figure 6). Starch percentage was high in seeds treated with 50 ppm (77.2%) compared to a percentage level of 70.8% of starch in untreated seeds. These results suggest that carbohydrate decomposition does not correlate to starch levels in NPs treated seeds, and this is most likely explained by the use of alternative carbohydrate sources. Another explanation is that the total carbohydrate content measured in the assay includes the composition of both the structural and the nonstructural carbohydrate in lettuce seeds.

Starch hydrolysis yields the monomers to supply the germinating seeds with the necessary nutrients and energy (Beck and Ziegler, 1989). Alpha amylases are the main crucial starch digesting enzymes that play an important role in seed germination. The role of Beta amylases in the germination of lettuce seed, or even other types of seeds, is not clearly studied in literature and their activities may be not a determining factor in seed germination.

**Figure 7:**
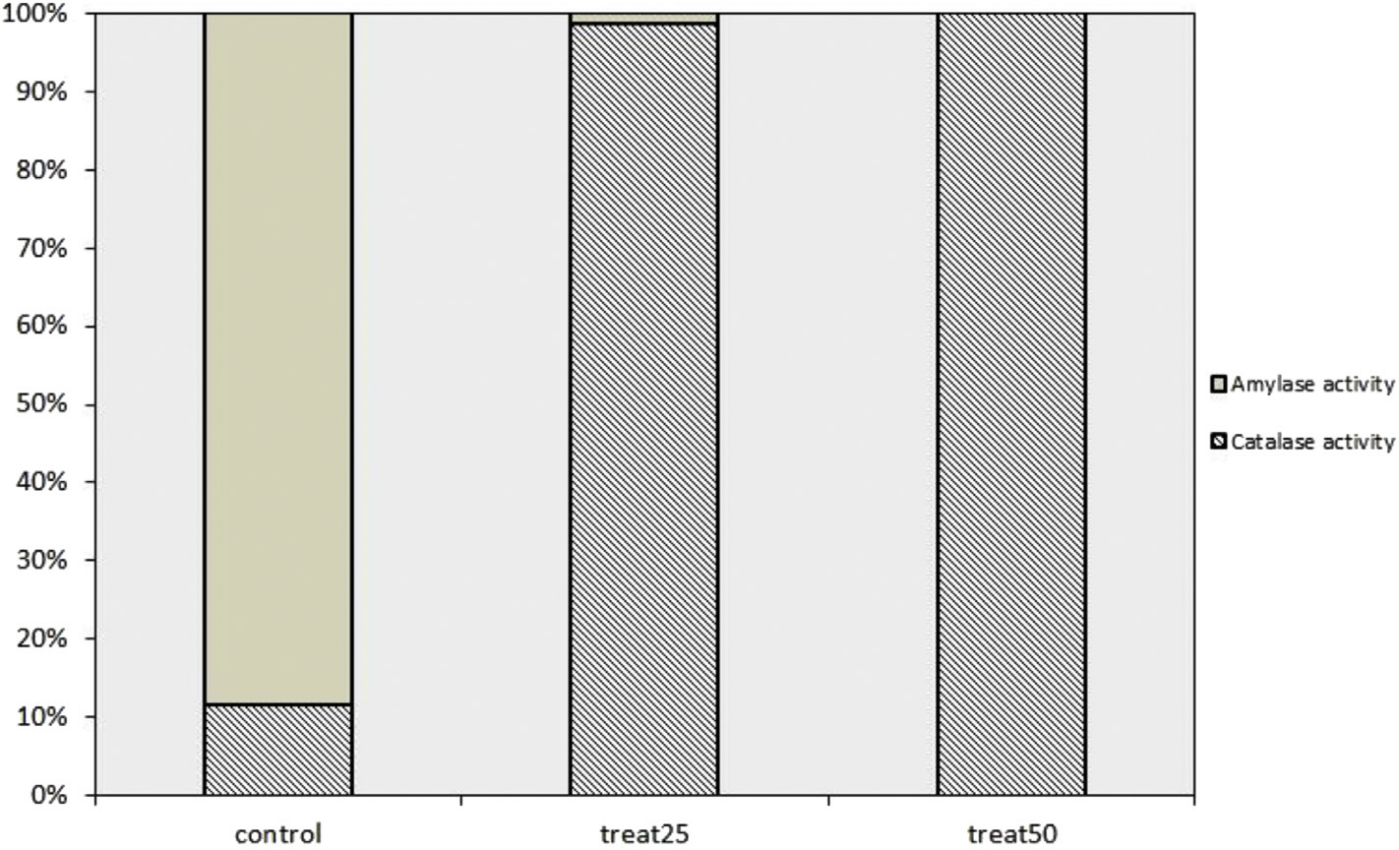
The relative activities of catalase and amylase enzymes in lettuce seeds treated with two concentrations of ZnO NPs; 25 ppm and 50 ppm. The total of each bar is 100 and the measured activity of each one of the enzymes is expressed as percentage of the respective totals.

High Starch content measured in treated seeds is in coincidence with the detectable low β amylase activity (Figure 7). The descending order of the detected activity of β amylase in seeds is: 1.6 U/g, 0.0426 U/g, 0.0 U/g for the untreated seeds, seeds treated with 25 ppm NPs and seeds treated with 50 ppm NPs respectively (Figure 7). This may be attributed to the lack of activation of amylases of imbibed seeds because of short term duration of the imbibition process that lasted for 3 h and which was not enough to activate the enzymes. This suggests that amylase activity increases gradually and its activation takes place over a longer period of reaction time.

In addition to amylolytic activity, alteration in the antioxidant status among the different imbibed seeds was investigated by detecting Catalase activity. ZnO NPs induced changes in catalase activity (Figure 7). Some results indicated that catalase activity is initially low during germination and increases during seedling establishment (Hite et al., 1999). ZnO NPs imbibed seeds exhibited high catalase activity (Figure 7) and according to literature there is an inverse relationship between H_2_O_2_ level and CAT activity in seed during their development. Increased levels of CAT means that there is low amount of H_2_O_2_ and the high protein levels might, on the basis of the current work, offer a rational explanation for the high measurement of CAT activity. The higher CAT activity is basically due to NPs modulating the enzymatic antioxidants in seeds, Moreover; many studies observed the ability of NPs to mimic the roles of antioxidant enzymes such as CAT. Plants with promoted antioxidants are more resistant to the oxidative conditions and less susceptible to stress challenging environment (Wei and Wang, 2013; Khan et al., 2017).

Germination and seedling emergence involves the utilization of the stored biomolecules such as carbohydrates and proteins because of the high demands for energy for the metabolism/synthesis of seed food reserves (Bewley and Black, 1994). Rupture of seed coat leads to emergence of seedling. Lettuce seeds imbibed in NPs showed accelerated and activated growth compared to the control untreated seeds. However the activity in seeds does not parallel water uptake or the decrease in amylase activity.

On the basis of the findings it is proposed that the increase in seedling length and biomass is due to some kind of *de novo* synthesis of protein. Based on the stimulated catalase enzyme activity and the suggested model of the action of zinc oxide nanoparticles is to act via zinc ion release that enter through seed coat. Once the ions are inside seeds they modulate protein synthesis and metabolism as well as carbohydrate content.

## 4 Conclusions

This comprehensive study analyzed suspensions of moderately polydisperse zinc oxide nanoparticles at concentrations of 25 ppm and 50 ppm for seed germination. Treated seeds were stored before germination; seed thus treated may be stored for some time after their treatment and still germinate in a greater way than untreated seeds. Nanoprimed seeds showed normal topography of the surface of seeds, no deformation was induced upon NPs treatment, and there was an increase in zinc level inside seeds, AAS confirm zinc uptake by seeds through ions release mechanism. An increase in the total protein levels indicates the *de novo* synthesis of enzymes that carry out later metabolizing reactions during visible germination. On other hand, total carbohydrates were relatively low in treated seeds, as a result of their utilization for energy production to meet the high energy demand for the primary cellular process in the primed seeds. Three hours imbibition, as an early stage of lettuce seed germination, was not enough for the activation of amylase activity and even the presence of NPs did not cause an early breakdown of stored starch.

Lettuce seeds treated with 25 ppm of ZnO NPs significantly enhanced lettuce seedling through the passage of zinc ions inside seeds resulting in powerful priming and the subsequent established visible germination. Lettuce seed treatment with zinc oxide nanoparticles for 3 h was enough to exert noticeable effect on the biomass and behavior of seeds.

## Declarations

### Author contribution statement

R. Rawashdeh: Conceived and designed the experiments; Performed the experiments; Analyzed and interpreted the data; Contributed reagents, materials, analysis tools or data; Wrote the paper.

A. Harb: Conceived and designed the experiments; Analyzed and interpreted the data; Contributed reagents, materials, analysis tools or data; Wrote the paper.

A. Al Hasan: Performed the experiments; Analyzed and interpreted the data; Wrote the paper.

### Funding statement

R. Rawashdeh was supported by The Dean of Scientific Research and Graduate Studies at 10.13039/501100006418Yarmouk University (13/2017).

### Competing interest statement

The authors declare no conflict of interest.

### Additional information

No additional information is available for this paper.

## Appendix A Supplementary data

Supplementary material _spl_Fig. S1_spl_ Supplementary material _spl_Fig. S1_spl_

Supplementary material _spl_Fig. S2_spl_ Supplementary material _spl_Fig. S2_spl_
