# Supplementary figures and images for: Biological interaction levels of zinc oxide nanoparticles; lettuce seeds as case study
Source: Heliyon. 2020 May 29;6(5):e03983. doi: 10.1016/j.heliyon.2020.e03983 (PMC7264067; doi:10.1016/j.heliyon.2020.e03983)

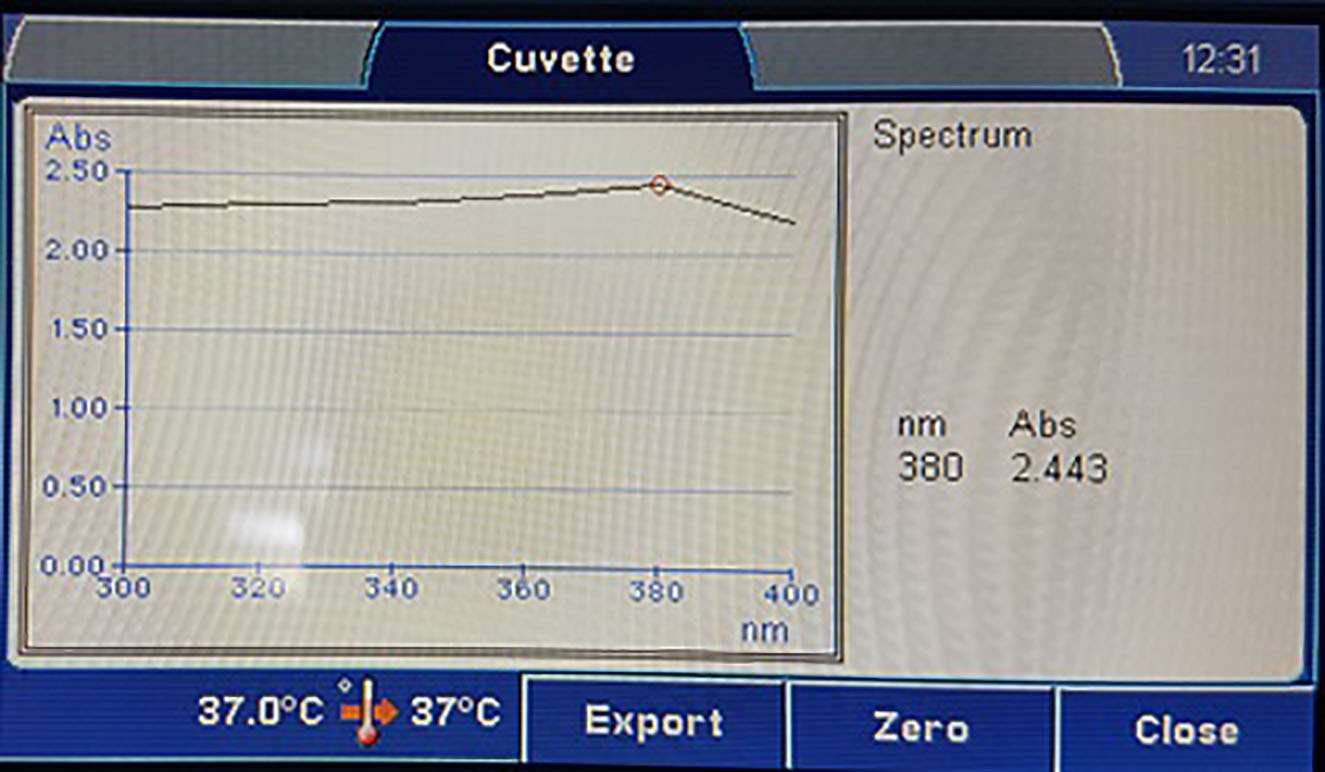


Supplementary material (Fig. S1). UV visible spectrum of Zinc oxide nanoparticles

Supplement: Supplementary material _spl_Fig. S1_spl_ [file mmc1.docx]
