# Supplementary material for: Biological interaction levels of zinc oxide nanoparticles; lettuce seeds as case study
Source: Heliyon. 2020 May 29;6(5):e03983. doi: 10.1016/j.heliyon.2020.e03983 (PMC7264067; doi:10.1016/j.heliyon.2020.e03983)
Supplement: Supplementary material _spl_Fig. S2_spl_ [file mmc2.docx]

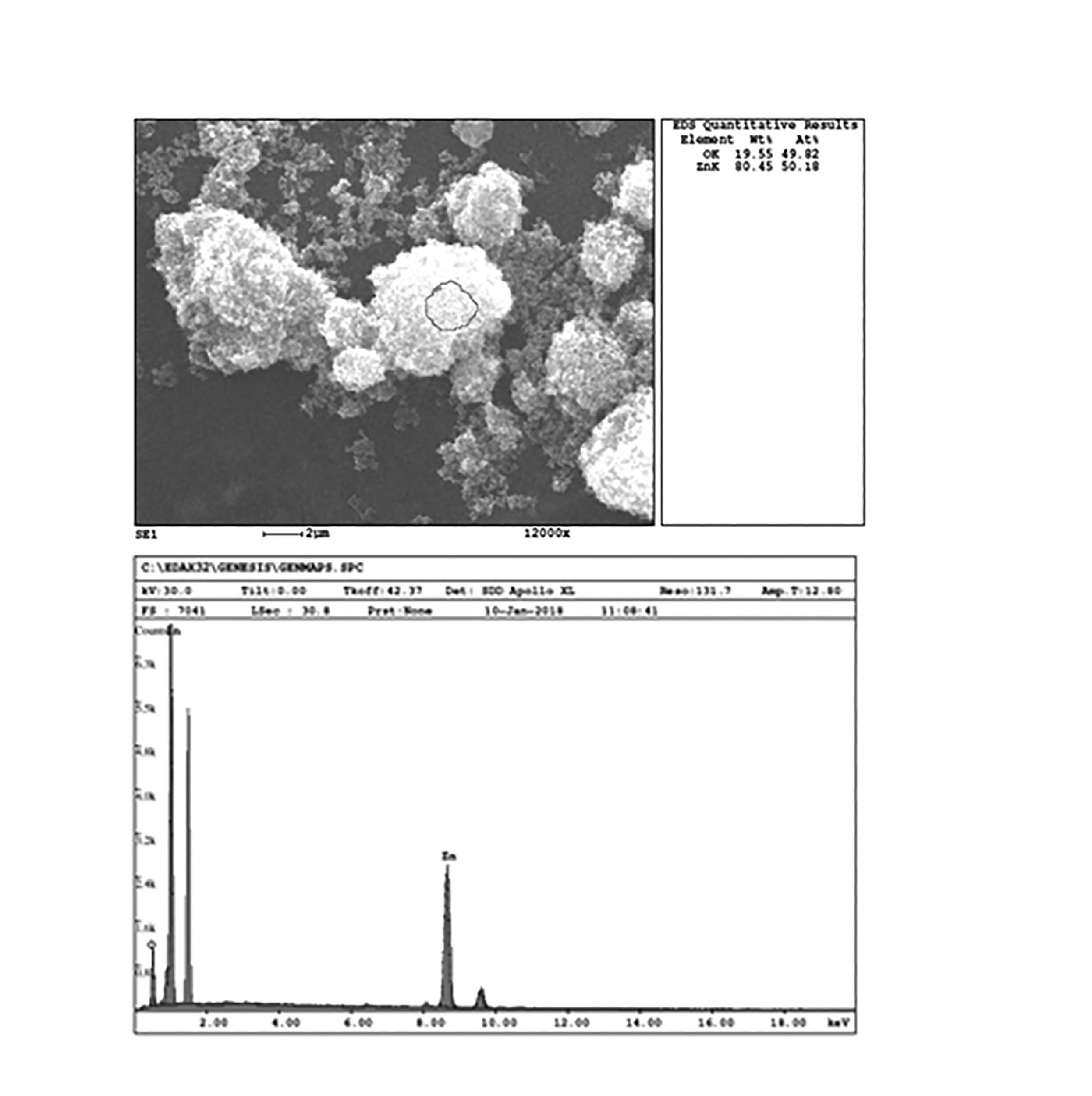


Supplementary material (Fig. S2). Energy dispersive x rays (EDX) for the analysis of the elemental composition of ZnO nanoparticles.
